# Evaluation of diastolic function by cardiac MRI in hypertrophic cardiomyopathy: validation of 3D model-based analysis of left ventricular filling curves

**DOI:** 10.1186/1532-429X-16-S1-P298

**Published:** 2014-01-16

**Authors:** Linda Chu, Celia P Corona-Villalobos, Neville Gai, David Bluemke, Marcelo Nacif, Alistair Young, Theodore Abraham, Ihab R Kamel, Stefan L Zimmerman

**Affiliations:** 1 Johns Hopkins University, Baltimore, Maryland, USA; 2 National Institute of Health, Bethesda, Maryland, USA; 3 Universidade Federal Fluminense, Niterói, Brazil; 4 University of Auckland, Auckland, New Zealand

## Background

Diastolic function (DF) is not routinely assessed by cardiac MR (CMR), but is critical for assessment of common disease states such as heart failure or hypertrophy. At present, special pulse sequences (tagging, phase contrast) are necessary for CMR for diastolic function assessment. The purpose of this study was to assess cine CMR volumetric assessment of DF using a 3D model-based analysis, in reference to 2D phase contrast and echocardiography (echo).

## Methods

In this IRB-approved retrospective study, consecutive subjects with HCM who underwent both echo and cardiac MRI from December 2011 to June 2013 were identified from the radiology database. Cine steady state free precession (SSFP) images (temporal resolution ~ 25 msec) were analyzed using a semiautomated 3D model-based analysis of left ventricular filling curve (CIM, Auckland MRI Research Group). Transmitral flow curves were measured from 2D phase contrast (PC) using dedicated software (Leonardo, Siemens). DF on echo was obtained from the medical records. Correlation of E/A ratio and E/E' ratio among CIM, 2D PC, and echo was calculated using Pearson's correlation coefficient. Differences in CIM E/A and E/E' ratios among DF groups were analyzed using ANOVA.

## Results

64 subjects were included; 49 males (76.6%) and 15 females (23.4%), with mean age of 53.3 years. CIM E/A and E/E' ratios were analyzable in 62 patients and 38 patients, respectively. DF grades on echo were: grade 0 (n = 16), grade 1 (n = 9), grade 2 (n = 29), grade 3 (n = 6), and not available (n = 4). Due to abnormal cardiac morphology of HCM patients, CIM software had minor difficulties with semiautomated contour tracking, which was easily adjustable by the user. CIM performed well in tracking of valve motion. There was strong correlation between E/A ratio of CIM and 2D PC (r = 0.799, p < 0.001) and between E/A ratio of CIM and echo (r = 0.726, p < 0.001). There was no significant correlation between E/E' ratio of CIM and 2D PC (r = 0.314, p = 0.296) and between E/E' ratio of CIM and echo (r = 0.078, p = 0.649), likely due to smaller sample size. There was significant difference in CIM E/A ratio among DF groups on one-way ANOVA (p < 0.001). Difference in CIM E/E' ratio among DF groups approached significance (p = 0.106).

## Conclusions

Semiautomated 3D model-based analysis of left ventricular filling curve is a promising technique for evaluation of DF in patients with hypertrophic cardiomyopathy, demonstrating good correlation with 2D phase contrast and echo in assessment of E/A ratio. DF can be measured accurately using routine cine SSFP images and semiautomated analysis of left ventricular filling curve, without the need for specialized pulse sequences.

## Funding

Linda Chu received research support from RSNA R&E Foundation Fellow Grant.

**Figure 1:**
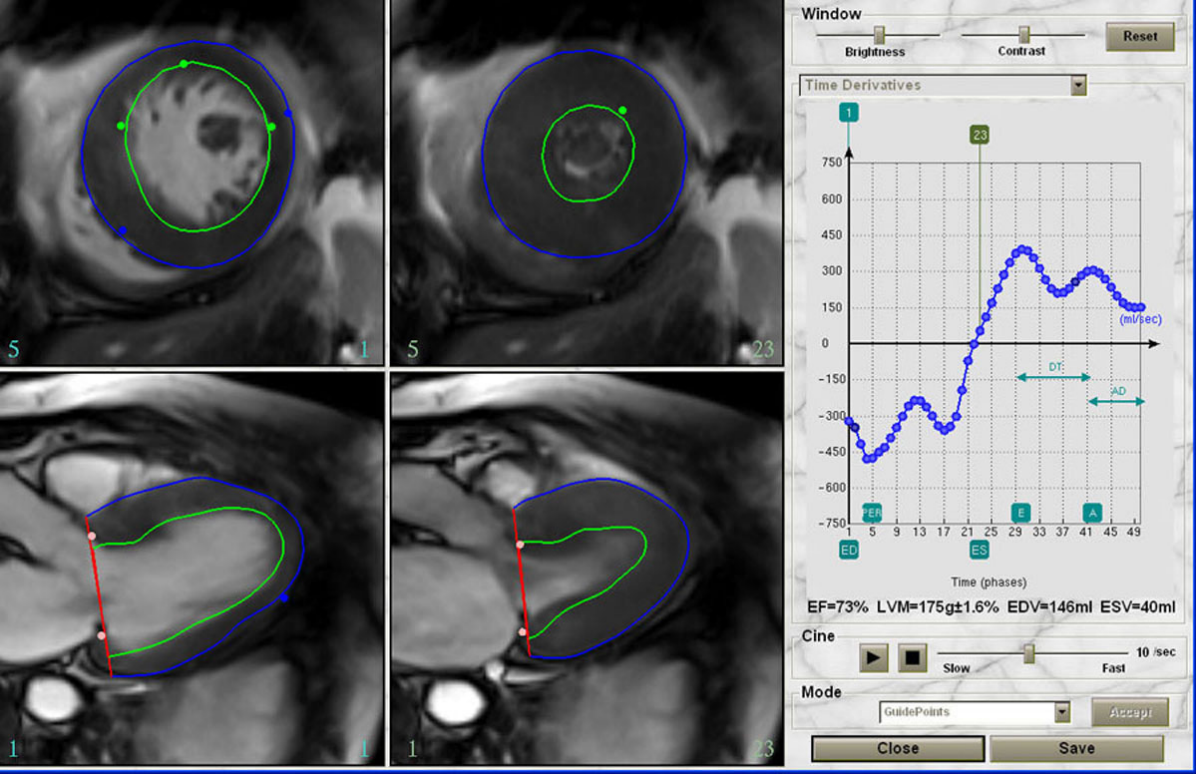
**Screenshot of Cardiac Imaging Modeling (CIM) analysis**. CIM program semi-automatically defines endocardial and epicardial contour throughout the cardiac cycle and creates the LV time-volume curve. Time derivatives of the LV time-volume curve such as early peak filling rate (E), atrial peak filling rate (A), deceleration time (DT), time to peak filling rate can be obtained semi-automatically with manual correction by the user.

